# A common dynamic prior for time in duration discrimination

**DOI:** 10.3758/s13423-021-01887-z

**Published:** 2021-03-04

**Authors:** Joost de Jong, Elkan G. Akyürek, Hedderik van Rijn

**Affiliations:** Department of Experimental Psychology, University of Groningen, Grote Kruisstraat 2/1, 9712 TS Groningen, the Netherlands

**Keywords:** Time perception, Bayesian modelling, Duration discrimination, Context effects

## Abstract

Estimation of time depends heavily on both global and local statistical context. Durations that are short relative to the global distribution are systematically overestimated; durations that are locally preceded by long durations are also overestimated. Context effects are prominent in duration discrimination tasks, where a standard duration and a comparison duration are presented on each trial. In this study, we compare and test two models that posit a dynamically updating internal reference that biases time estimation on global and local scales in duration discrimination tasks. The internal reference model suggests that the internal reference operates during postperceptual stages and only interacts with the first presented duration. In contrast, a Bayesian account of time estimation implies that any perceived duration updates the internal reference and therefore interacts with both the first and second presented duration. We implemented both models and tested their predictions in a duration discrimination task where the standard duration varied from trial to trial. Our results are in line with a Bayesian perspective on time estimation. First, the standard systematically biased estimation of the comparison, such that shorter standards increased the likelihood of reporting that the comparison was shorter. Second, both the previous standard and comparison systematically biased time estimation of subsequent trials in the same direction. Third, more precise observers showed smaller biases. In sum, our findings suggest a common dynamic prior for time that is updated by each perceived duration and where the relative weighting of old and new observations is determined by their relative precision.

Time estimation is something we do naturally on a daily basis; when we prepare a meal, take turns in a conversation, and in countless other instances. The estimates we make are not absolute, however, as was first demonstrated by von Vierordt (1868). Time estimation is heavily influenced by statistical context. Both the overall distribution of durations (global context) and immediately preceding durations (local context) bias temporal estimates (e.g., Acerbi, Wolpert, & Vijayakumar, 2012; Jones & Mcauley, 2005; Shi, Church, & Meck, 2013; Taatgen & van Rijn, 2011; van Rijn, 2016). In the case of global context effects, temporal estimates show a regression to the mean of the distribution of durations that were previously encountered. Local context effects refer to the current estimate being pulled towards immediately preceding durations, so-called *n*−1 effects. While both effects highlight context effects at different time scales, they are nevertheless thought to reflect common underlying processes (Bausenhart, Bratzke, & Ulrich, 2016).

Many different paradigms have been used to study context effects in time estimation. We will focus, however, on one particular psychophysical method: duration discrimination. In duration discrimination, a participant is presented with two subsequent durations per trial: a fixed standard duration (S) and a comparison duration (C), which differs from the standard. Participants are asked whether the first or second stimulus was longer. Temporal accuracy can be assessed by manipulating the difference between S and C and fitting a psychometric curve. The slope of the resulting curve reflects temporal precision, whereas the location where the curve crosses the 50% line (Point-of-Subjective-Equality; PSE) reflects biases. Results from duration estimation tasks have shown that the order in which S and C are presented matters. Temporal precision is higher for the order <sc> than for <cs> (Dyjas, Bausenhart, & Ulrich, 2012). This order effect has been referred to as a Type B effect, where Type A refers to effects of stimulus order on bias (Lapid, Ulrich, & Rammsayer, 2008). The Type B effect has been taken to suggest that the global context in which durations are embedded influences duration estimation (Bausenhart et al., 2016). The global distribution of durations for the first duration is more variable for order <cs>, producing less precise temporal judgements. Moreover, only when the stimulus order is <cs> does the comparison on the previous trial (C_*n−*1_) systematically bias duration estimates, demonstrating local context effects.

Several models have been proposed to explain context effects in time estimation, but we will discuss two main theoretical approaches: The internal reference model (IRM; Dyjas et al., 2012) and Bayesian models (Jazayeri & Shadlen, 2010; Shi et al., 2013; Wiener, Thompson, & Coslett, 2014). Originally proposed to account for Type B effects in duration discrimination (Lapid et al., 2008), IRM proposes that an internal reference represents a moving average of previous durations. IRM assumes that, on each trial, the internal reference is only updated by the first presented duration. According to IRM, the fact that the first duration is incorporated into the internal reference, whereas the second duration is not, is what causes Type B effects: With stimulus order <sc>, only the *fixed* standard is incorporated into the internal reference, making for a less variable internal reference and therefore higher precision. In contrast, with stimulus order <cs>, only the *variable* comparison is incorporated into the internal reference, making for a more variable reference, resulting in lower precision. A second major prediction of IRM is that the local context effect of the previous comparison (C_*n−*1_) depends on the stimulus order. More specifically, C_*n−*1_ should have no effect on the position of the psychometric curve when the stimulus order is <sc>, since the second stimulus is never incorporated into the internal reference. However, C_*n−*1_ should influence the position of the psychometric curve when the stimulus order is <cs>. Indeed, this is what Dyjas et al. (2012) found: a significant local context effect was only observed with stimulus order <sc>. Dyjas et al. (2012) also considered an alternative version of IRM where both the first *and* second stimulus are incorporated in the internal reference. It was noted that this alternative would account for their results equally well as IRM proper, but the ‘first-only’ version was preferred, because it was considered less complex.

Despite their overall similarity with IRM, Bayesian models provide a different perspective on context effects in time estimation (Shi et al., 2013). While both IRM and Bayesian models consider a time estimate to be the product of a weighted average of an internal reference (i.e., the prior) and sensory information (i.e., the likelihood), they disagree on how these weights are determined. In IRM, the relative weight (*g*) is a free parameter that may differ between experimental conditions (Dyjas, Bausenhart, & Ulrich, 2014). Also, IRM does not suggest a rigorous functional interpretation of this internal reference. After all, the internal reference may degrade performance, depending on the order of stimuli. In contrast, Bayesian models weigh the likelihood and prior by their relative precision on each individual trial, giving more weight to the more precise source of temporal information. This results in a statistically optimal time estimate. Thus, Bayesian accounts of time estimation suggest a functional explanation for context effects: Time estimates are systematically biased for the purpose of optimal estimation under noisy conditions. Shi et al. (2013) point out that the Bayesian implementation of the updating process of IRM is referred to as a Kalman filter, a method which has recently been successfully used to explain a wide variety of context effects in magnitude estimation (Petzschner, Glasauer, & Stephan, 2015).

Another important difference between IRM and Bayesian models is the locus of context effects. IRM strongly implies that temporal representations are influenced by context during postperceptual stages, given that the internal reference does not influence the representation of the second stimulus (which is perceived nevertheless). As a result, context effects are explained by the first duration on the previous trial. In contrast, Bayesian models suggest that temporal representations are already sculpted by context at perceptual stages. Bayesian models of magnitude estimation assume that the product of combining sensory input (i.e., the likelihood) and memory (i.e., the prior) results in the posterior, which *is* the percept (Petzschner et al., 2015). In such a model, the common prior is dynamically updated by each duration. It follows that in duration discrimination tasks, not only the first but also the second duration should systematically bias subsequent time estimates in the same direction. A further consequence of such a model is that the first duration also systematically biases perception of the second duration.

Here, we implemented both IRM and a Kalman filter and generated predictions from these models. Subsequently, we performed a duration estimation experiment to compare and contrast the IRM and Bayesian models of time estimation with regard to local and global context effects. Our empirical findings are in line with a Bayesian model where a dynamic prior is sequentially updated by each duration. In a duration discrimination task with a variable standard on each trial (the roving-standard task; Allan & Gerhardt, 2001), we observed three effects. First, we found systematic biases for each standard, in line with the standard influencing the comparison. Second, both the first and second duration biased subsequent time estimates in the same direction. Third, more precise observers showed smaller biases. These findings suggest that both global and local context effects result from a common dynamic prior that is sequentially updated by both the first and second duration.

## Methods

### Participants

A total of 47 students (29 females; mean age 20.6 years) at the University of Groningen participated in exchange for partial course credit. Participants gave informed consent before the experiment. Based on the predefined performance criterion (see Procedure section), one participant was excluded from further analysis.

### Apparatus and stimuli

Stimulus generation and presentation were controlled by Psychtoolbox (Brainard, 1997). We used a 19-in. CRT screen, an Iiyama Prolite G2773HS-GB1, with a resolution of 1,280 × 1,024 pixels, running at 100 Hz. Participants were seated in a sound-attenuated room with dimmed lights approximately 60 cm from the screen. A grey background was maintained during the entire experiment. A black fixation dot was presented throughout each trial. Feedback sounds were brief (150 ms) pure tones; high (1000 Hz) for correct and low (200 Hz) for incorrect responses. Stimuli were white circles, presented in the center of the screen with a diameter of 6.69°.

### Procedure

**Fig. 1:**
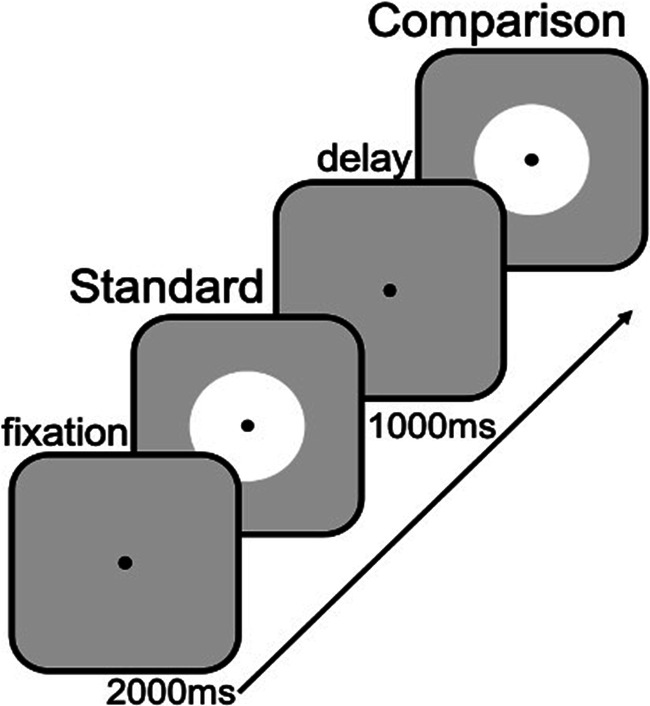
Trial structure. A fixation dot was presented for 2 s at the start of every trial. Then, the standard was presented, which had a duration of 0.3, 0.6, 1.2 or 2.4 s. After a delay of 1s, the comparison was presented, which was either shorter or longer than the standard. Participants had to indicate whether the comparison duration was shorter (press ‘c’) or longer (press ‘m’) than the standard

After four practice trials (one for each standard duration, in random order), participants completed a total of 200 trials of a duration discrimination task (see Fig. 1). On each trial, the duration of a standard (S) duration (always presented first) was compared with a comparison (C) duration. At the start of each trial, a black fixation dot was presented centered on the screen, which was present throughout the entire trial. After 2 seconds, the standard was presented, which had a duration of 0.3, 0.6, 1.2, or 2.4 seconds (randomly sampled without replacement). Then, after a delay of 1 second, C was presented, which was either shorter or longer than S. The difference in duration between S and C is referred to as Δd, which is a proportion of S. When C was shorter than S, the comparison duration equaled $\frac{S}{1+\varDelta d}$; when it was longer, its duration equaled *S* ∗ (1 + *Δd*). Participants indicated whether C was shorter (key ‘c’) or longer (key ‘m’) than S. Participants also received auditory feedback after their response: a brief high tone for correct, and a brief low tone for incorrect responses. All combinations of S and longer/shorter C were presented equally often (50% of the comparisons were longer, 50% was shorter, across standards) and in randomized order.

The difference in duration between the S and C (Δd, in proportion to S) was varied with an adaptive staircase procedure throughout the experiment. A transformed-rule up and down was used. When participants gave three consecutive correct responses, Δd was decreased, and it was increased when they made a single error. This rule approximates a performance level of 79.4% correct (Levitt, 1971). The starting value of Δd was 0.6 with a step size of 0.05 and a minimum of 0.05. When participants reached a Δd of more than 1, they were excluded from any further analysis (see Participants section).

### Analysis

Generalized linear mixed models (GLMMs) were fitted with the *lme4* package (Version: 1.1-19; Bates, Mächler, Bolker, & Walker, 2015) using the ‘nlminb’ optimizer from the *optimx* package (Version 2018-7.10; Nash & Varadhan, 2011). Mixed-effect models are more powerful than the traditional approach of aggregating data on a subject level, since it takes into account subject-level variability (Moscatelli, Mezzetti, & Lacquaniti, 2012). The data were fitted to normal cumulative psychometric functions with the ‘probit’ function. We centered S around the geometric mean (849 ms) to make the results more interpretable. Assuming that the geometric mean represents the center of the duration distribution, this will produce an overall intercept of zero. In the GLMMs, ‘subject’ was always included as a random intercept. We sequentially added fixed and their associated random effects to the GLMM. To quantify evidence for more complex models over simpler ones, Bayes factors were approximated with the Bayesian Information Criterion (BIC) values of the GLMMs (Wagenmakers, 2007):

1 $$ {BF}_{01}=\exp \left(\frac{{\Delta BIC}_{10}}{2}\right) $$

In order to quantify the relationship between bias and precision, we estimated the effect of standard and the average slope for each participant individually in a GLM. Then, we computed to nonparametric Spearman correlation coefficients and associated 95% confidence intervals, using the *z*-transformation method implemented in the *psych* package (Version 2.0.12; Revelle, 2020).

### Modelling

In order to formalize the differences between IRM and the Kalman filter, we implemented both in R (R Core Team, 2018). We did not perform extensive optimization routines to fit each model to the data, since we only want to demonstrate what each model predicts given a reasonable set of fixed parameters. We wanted to ensure that both models have identical inputs and similar rules for determining outputs, so that differences in model predictions can be attributed to the internal workings of each model. Inputs (*x*_*m*_) were logarithmically transformed durations (*d*), as used in the experiment, perturbed by gaussian noise (*n*_*m*_):

2 $$ {x}_m=\ln (d)+{n}_m $$

with $p\left({n}_m\right)=N\left(0,{\sigma}_m^2\right)$. Here, ${\sigma}_m^2$ determines the noisiness of the sensory input. Put differently, $1/{\sigma}_m^2$ is the precision of sensory input. In order to simulate data for individual subjects with different precision, we randomly selected values for *σ*_*m*, *i*_ from a truncated normal distribution where the mean corresponds to *σ*_*m*_. Importantly, these input parameters were not free parameters, since they were fixed across different models. We found that *σ*_*m*_ = 0.2 provided a reasonable fit for the aggregate results. We simulated data from each model for 200 subjects with 840 trials each, matching the random order of stimulus presentations to the real participants. We did not run a staircase procedure on simulated subjects, but instead presented combinations of S and Δd in random order without replacement.

Outputs of the models (responding ‘longer’ or ‘shorter’) were determined by comparing the representations of *S* and *C* produced by each model. If *C > S*, the model responds, ‘comparison longer’; if *C < S*, ‘comparison shorter.’ In the case of IRM, *S* and *C* were the internal references that resulted from perceiving the standard and comparison. In the case of the Kalman filter, *S* and *C* were the means of the priors that resulted from perceiving the standard and comparison.

#### Kalman filter

The Kalman filter is a Bayesian model, which assumes that subjects maintain and update a prior, which represents the distribution of previously observed durations. We base our implementation of the Kalman filter on Glasauer and Shi (2018), and Petzschner and Glasauer (2011). Instead of representing durations with only a single value, the Kalman filter also represents the uncertainty associated with that representation. The prior is modelled as a Gaussian distribution: $N\left({\mu}_p,{\sigma}_p^2\right)$. When a duration, indexed by *n*, is sensed*,* it is represented by the likelihood function $p\left({x}_{m,n}\right)\sim N\left({x}_{m,n},{\sigma}_m^2\right)$. When a duration is estimated, the prior is updated through a weighted average of the previous prior distribution and the currently sensed likelihood. The weight of the new observation is called the Kalman gain (*k*):

3 $$ {k}_n=\frac{\sigma_{p,n-1}^2+q}{\sigma_{p,n-1}^2+q+{\sigma}_m^2} $$

As can be seen, the weight of the new observation is determined by the relative uncertainty of the previous prior ${\sigma}_{p,n-1}^2$ and uncertainty of the current likelihood ${\sigma}_m^2$. When the uncertainty of the current sensory observation (likelihood) is large relative to the uncertainty of the prior, *k* will be small (for a method to empirically estimate the Kalman gain over time, see Berniker, Voss, & Kording, 2010). The Kalman gain is further determined by process variance *q*, which reflects that the observer assumes a prior that fluctuates randomly over time: *μ*_*p*, *n*_~*μ*_*p*, *n* − 1_ + *N*(0, *q*). In other words, there is always a level of uncertainty involved in representing the non-static prior, which is determined by process variance *q*. This is an important assumption, since the variance of the prior ${\sigma}_p^2$ is updated as follows:

4 $$ {\sigma}_{p,n}^2={k}_n\ast {\sigma}_m^2 $$

We can see from Equations 3 and 4 that, if *q* = 0, and ${\sigma}_m^2$ is constant, *k* would continually decrease alongside ${\sigma}_p^2$, ensuring that new observations are unable to change the prior, resulting in an overly rigid representation of stimulus history. The prior mean (*μ*_*p*_) is updated as follows:

5 $$ {\mu}_{p,n}=\left(1-{k}_n\right)\ast {\mu}_{p,n-1}+{k}_n\ast {x}_{m,n} $$

In order to use the Kalman filter for duration discrimination, we assume that subjects use the updated prior for both the first and second duration and compare their means. For all simulations, we used *q* = 0.9. We chose this value because this produced an average *k* around 0.85 across simulated subjects. This, in turn, ensured that the parameters of the Kalman filter and IRM are comparable, since the Kalman gain determines weight on new observations, and *g* determines the weight on the internal reference (i.e., *k* = 1 − *g*). It should be noted that, since the variance of the likelihood function (${\sigma}_m^2$) varies between subjects, *k* also varies between subjects. When ${\sigma}_m^2$ is high (low), *k* will be low (high), and the prior will have a more (less) pronounced influence in the form of global and local context effects. In other words, more precise subjects will have smaller context effects.

#### Internal reference model

The internal reference model (IRM; Dyjas et al., 2012) assumes that subjects maintain and update an internal reference, which represents a geometric moving average of previously observed durations. When a duration *x*_*m,n*_, indexed by *n*, the internal reference *I*_*n*_ is updated through a weighted average of the previous internal reference (*I*_*n*−1_) and the currently observed duration *x*_*m*_:

6 $$ {I}_n=g\ast {I}_{n-1}+\left(g-1\right)\ast {x}_{m,n} $$

where *g*, 0 ≤ *g* < 1 is the constant weight on *I*_*n*−1_. In effect, *g* controls a trade-off between having a stable internal reference (high *g*) and quickly adapting to new durations (low *g*). Dyjas et al. (2012) describe two different versions of IRM that can be used to explain performance in duration discrimination tasks. In the first version, which we will refer to as IRM1, only the first duration updates the internal reference. This internal reference, in turn, is compared with the observed second duration, which does not update the internal reference. In the second version (IRM2), both durations update the internal reference and both internal references are compared with generate a decision about whether the second stimulus was longer or shorter than the first. For all simulations, we use *g* = 0.15. All data, materials, code and supplemental materials are available on the Open Science Framework (osf.io/hu43y).

## Results

The overall performance (80.6% correct) suggested that the staircase procedure was successful. Further, the standard deviation of Δd for each participant decreased after approximately 60 trials and remained relatively stable after, suggesting that the staircase procedure converged. First, a binomial probit generalized linear model (GLM) was fitted with ‘C longer response’ as dependent variable and Δd as predictor. The model significantly improved when first adding random intercepts (BF_01_ < 0.001), and then random slopes (BF_01_ < 0.001) for each subject. This suggested that there was a significant amount of interindividual variability in both precision and the location of the psychometric curves. In all following GLMMs, random intercept and slope for each subject were included.

**Fig. 2:**
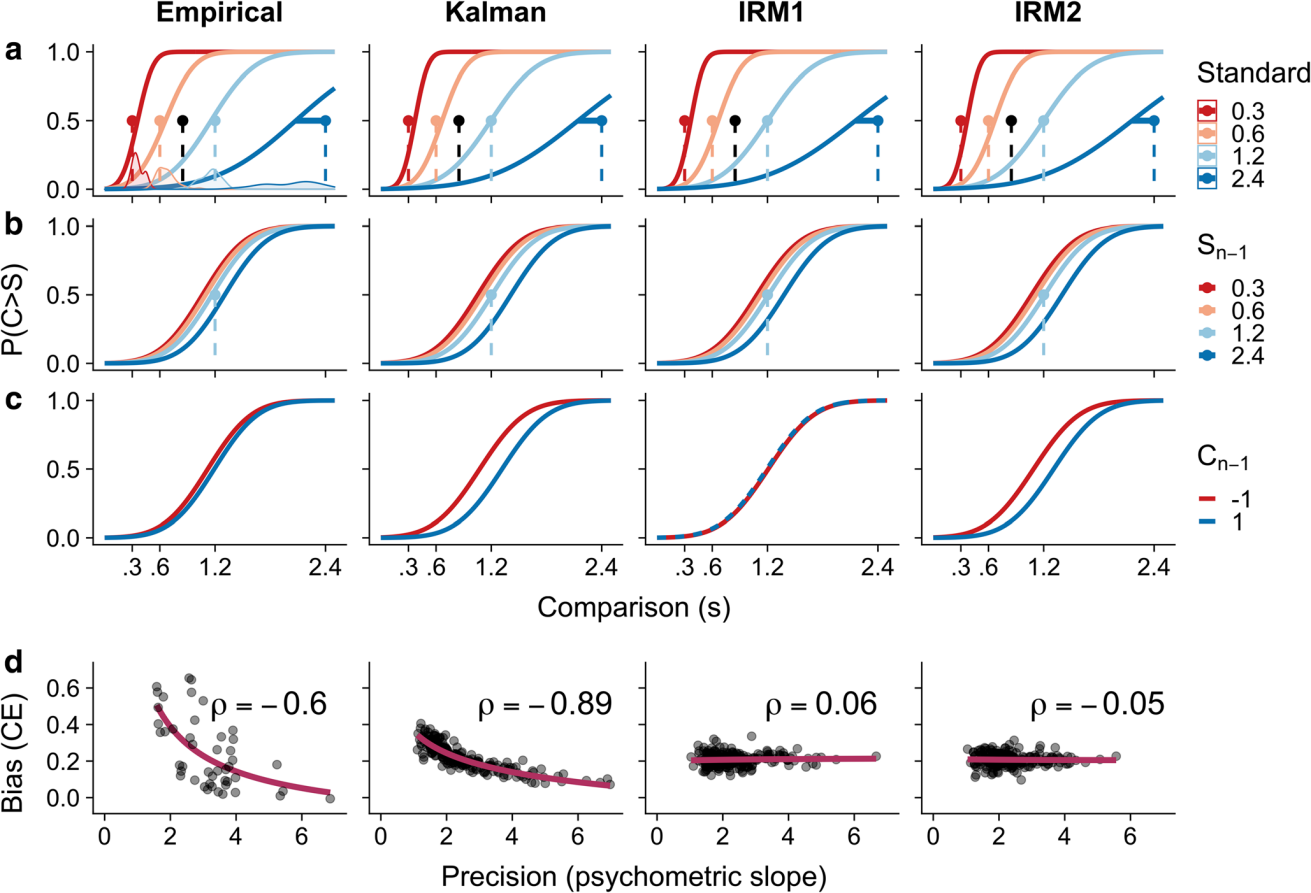
Fitted psychometric curves for empirical data and each model. **a** The effect of current standard on bias. Dashed lines and dots represent the standard durations and geometric mean, which is connected with a horizontal line to the point-of-subjective-equality (PSE). Density plots represent the subject-to-subject variability in PSE estimates for each standard duration. For psychometric curves, the *x*-axis represents the comparison duration and the *y*-axis represents probability of ‘C longer’ response. **b** Statistical estimates for the effect of S_*n*−1_ on PSE, plotted for S_*n*_ = 1.2 s. **c** Statistical estimates for effect of C_*n*−1_ on PSE, where ‘−1’ indicates that C_*n*−1_ was twice as short as S_*n*−1_ and ‘1’ indicates that it was twice as long. These values for C_*n*−1_ were chosen for illustrative purposes, since absolute values of Δd varied between participants, but absolute values of Δd were constrained to be less than or equal to 1. Also, these values ensure that these plots are on a similar scale as those for S_*n*−1_. **d** Estimated subject-to-subject Spearman correlation coefficients (ρ) from GLMs between bias (constant error; coefficient for S_*n*_) and precision (psychometric slope; coefficient for Δd). To demonstrate the functional form of this relationship, red lines are fitted power functions. (Color figures online)

When ‘S’ was added as a fixed effect, the model significantly improved (β = 0.49, BF_01_ < 0.001), suggesting that, overall, longer standards had a higher probability of ‘longer response’ (see Fig. 2a). At first sight, this might resemble a global context effect, given the regression to the overall distribution of durations. This would implicitly assume, however, that only S is affected by global context, whereas C is not. Alternatively, this result can be interpreted as an influence of S_*n*_ on C_*n*_. While the distribution of durations that precedes S_*n*_ spans the complete set of durations, the distribution preceding C_*n*_ is always biased, since C_*n*_ is always a multiple (between 0.5 and 2) of S_*n*_. As a consequence of this imbalanced local context, a short S_*n*_ will bias C_*n*_, such that the proportion of ‘C longer’ responses decreases, and vice versa.

Further, when an interaction between S and Δd was added, the model significantly improved (β = 0.42, BF_01_ < 0.001), which suggested that longer standards had steeper normalized slopes, reflecting a lower Weber fraction for higher durations. This violated the scalar property of time, which assumes that the standard deviation of time estimates scales linearly with the mean time estimate. However, we did not explicitly instruct participants not to count, which may increase sensitivity for durations longer than one second (Hinton & Rao, 2004). Further, when random slopes for ‘standard duration’ and its interaction with Δd were added, the model significantly improved (BF_01_ < 0.001), indicating that there was substantial interindividual variability in violating the scalar property.

Before assessing the role of S_*n*−1_ and C_*n−*1_, we first controlled for any confounding effects of feedback on the previous trial. Accuracy on the previous trial was coded as follows: −1 (incorrect: actual duration ‘shorter’), 0 (correct), and 1 (incorrect: actual duration ‘longer’). When we added previous accuracy, the model significantly improved (β = 0.151, BF_01_ = 0.030), suggesting participants incorporated feedback. More specifically, when the negative feedback indicated that C_*n−*1_ was actually longer, this increased the probability of responding ‘C longer’ on the current trial. We cannot conclusively show that this in fact improved performance, however, this result is compatible with Bayesian integration, where sources of information are used to bias responses and, in turn, improve performance. The model improved significantly when we added S_*n*−1_ (β = −0.197, BF_01_ < 0.001), suggesting that the previous standard assimilated time estimates on the current trial. When S_*n*−1_ was short, participants tended to respond ‘C longer’ more often, suggesting the S_*n*_ was assimilated by S_*n*−1_ (see Fig. 2b). Crucially, when adding C_*n−*1_, which was coded as Δd_*n−*1_ to prevent collinearity, the model further improved (β = −0.186, BF_01_ = 0.026). In line with Bayesian models, this suggests that both the first and second stimulus on the previous trial biased time estimates in the same direction (see Fig. 2c). The model improved when we included durations from S_*n–*2_ (β = −0.035, BF_01_ < 0.001), but not from C_*n–*2_ (β = 0.032, BF_01_ = 71.549), suggesting that influences from local context extend beyond the immediately preceding trial. However, the model did not improve when we added S_*n–*3_ (β = −0.027, BF_01_ = 12.306). Due to model convergence issues, we refrained from including random effects for durations and accuracy on previous trials.

Individual differences in precision and bias were also estimated by fitting a GLM with all fixed effects from the best fitting GLMM for each participant. From these fits, we estimated the precision and bias and computed the nonparametric Spearman correlation coefficient. In line with a Bayesian model of temporal estimation, our best fitting model suggested a high negative correlation, (*ρ*(44) = 0.60, 95 % *CI*[−0.76, −0.38], *p* < 0.001), between individual estimates in precision (coefficient for Δd) and the bias (coefficient for S). This suggests that participants with high precision had a weaker context effect (see Fig. 2d).

We also generated data from the Kalman filter, IRM1, and IRM2 and plot their results alongside the empirical results. As expected, all models capture global context effects and the effect of S_*n*−1_. In contrast to IRM2, IRM1 was unable to account for effects of C_*n*−1_, since that duration is not incorporated into the internal reference. However, neither IRM1, (*ρ*(198) = 0.06, 95 % *CI*[−0.08,  0.20], *p* = 0.40), or IRM2, (*ρ*(198) =  − 0.05, 95 % *CI*[−0.19,  0.08], *p* = 0.44), can account for the inverse relationship between precision and bias. In contrast, the Kalman filter can account for all three major empirical findings, (*ρ*(198) =  − 0.89, 95 % *CI*[−0.92, −0.86], *p* < 0.001). It should be noted that IRM2 and the Kalman filter overestimate the effect of C_*n*−1_, possibly due to a different actual weighting for standard and comparison durations.

## Discussion

Time estimation is shaped by statistical context. Systematic biases in time estimation due to global and local context are well-documented phenomena (van Rijn, 2016), however, it is not clear how or why they occur. IRM proposes that global and local context effects arise from an internal reference that is continually updated by either both durations or only the first one. Contrary to intuition, systematic biases in time perception may not reflect errors, but rather mechanisms that attempt to minimize error (Shi et al., 2013). In line with a Bayesian perspective on time estimation, we report three findings. First, time estimates were influenced by global context: relatively short standards resulted in underestimation of the comparison, and vice versa for long durations. This finding implies that the perception of the standard biases the perception of the comparison. In other words, the posterior that results from perceiving the standard serves as the prior for perceiving the comparison. In contrast, IRM1 would suggest that, since only the first duration is influenced by the global context, this should result in overestimation of short standards (and therefore underestimation of the comparison). Second, time estimates were influenced by local context: both durations in a discrimination task influenced time estimates on subsequent trials. This finding strongly suggests that durations are dynamically integrated into a common prior, regardless of their ordinal position in a duration discrimination task. Notably, contrary to our findings, IRM1 would predict that only the first duration should determine local context effects. Third, precision and bias were negatively correlated on a subject-by-subject basis, suggesting that more precise observers give more weight to current sensory information than priors. It should be noted that IRM2, where both stimuli are integrated into, and influenced by, the internal reference can also account for global and local context effects. However, it is unable to account for the relationship between precision and bias, since it does not take into account the precision of sensory observations and the internal reference in determining the relative weight of these sources of information.

One may wonder to what degree IRM is actually a Bayesian model of perception (or approximates one), given that it weighs current sensory inputs and prior information in a systematic fashion. Indeed, models that incorporate previous trials in the estimation process may approximate Bayesian inference (e.g., Raviv, Ahissar, & Loewenstein, 2012). Further, these models account for the effects of previous trials, which Bayesian models with a static prior are unable to do. However, as we have shown, IRM and the Kalman filter make different predictions in some important respects. Furthermore, IRM lacks some of the most important theoretical commitments that Bayesian models have. For instance, IRM can be adapted to account for effects of the second duration or lack thereof, while the Kalman filter is considerably less flexible. Bayesian models of perception are committed to the idea that all perceived stimuli are subjected to the influence previous observations. A similar argument holds for the relationship between precision and bias. IRM could make several assumptions about how precision and *g* are related in order to explain the data. In contrast, Bayesian models of perception are inherently committed to the idea that relative influence of the prior and likelihood are weighted according to their relative precision. Therefore, in the context of the current study, IRM could predict more different patterns of data (i.e., is less falsifiable; Roberts & Pashler, 2000) than the Kalman filter.

It seems unlikely that the observed effect of the C_*n*−1_ was solely due to sequential decision biases or integration of feedback on performance. Here, unlike effects of stimulus order on bias (time-order effects), sequential decision biases refer to the tendency of observers to give the same response as on the previous trial when uncertainty is high. Given the relatively constant high performance of our participants (around 80%), a decision bias would predict a positive effect of the previous comparison on the ‘comparison longer’ response; but we found the opposite. Also, our models corrected for influences of feedback on previous trials, such that the coefficient for C_*n*−1_ reflected context effects when the previous response was correct. These results are compatible with Wiener et al. (2014), who found that both decision biases and assimilative biases exist in visual duration estimation. Thus, in our experiment, assimilative biases may have been stronger than any existing decision biases.

Our findings also point to some important questions and avenues for future research. The relative contribution of the first and second stimulus could not be estimated with our current analysis. Therefore, an extensive modelling effort would be needed to reliably estimate the magnitude of context effects. More importantly, these estimates can be used to further test some crucial predictions of both IRM and Bayesian models. For instance, the magnitude of context effects in Bayesian models are solely due to the uncertainty of the prior and the observation. In IRM, however, the magnitude of context effects depends on *g*, which may be influenced by various cognitive processes, such as attention (Dyjas et al., 2014). Therefore, a more unifying account of context effects may predict that attention increases the precision of observations (likelihood) and therefore decreases the IRM-weight on observations (1-*g*). Indeed, integrating models of time estimation with established models of cognition has produced quantitative predictions that can arbitrate between competing models (Taatgen, van Rijn, & Anderson, 2007). This would thus seem a promising avenue for further research.

## References

[CR1] Acerbi L, Wolpert DM, Vijayakumar S (2012). Internal representations of temporal statistics and feedback calibrate motor-sensory interval timing. PLoS Computational Biology.

[CR2] Allan LG, Gerhardt K (2001). Temporal bisection with trial referents. Perception & Psychophysics.

[CR3] Bates D, Mächler M, Bolker B, Walker S (2015). Fitting linear mixed-effects models using lme4. Journal of Statistical Software.

[CR4] Bausenhart KM, Bratzke D, Ulrich R (2016). Formation and representation of temporal reference information. Current Opinion in Behavioral Sciences.

[CR5] Berniker M, Voss M, Kording K (2010). Learning priors for Bayesian computations in the nervous system. PLoS ONE.

[CR6] Brainard DH (1997). The Psychophysics Toolbox. Spatial Vision.

[CR7] Dyjas O, Bausenhart KM, Ulrich R (2012). Trial-by-trial updating of an internal reference in discrimination tasks: Evidence from effects of stimulus order and trial sequence. Attention, Perception, & Psychophysics.

[CR8] Dyjas O, Bausenhart KM, Ulrich R (2014). Effects of stimulus order on duration discrimination sensitivity are under attentional control. Journal of Experimental Psychology: Human Perception and Performance.

[CR9] Glasauer, S., & Shi, Z. (2018). 150 years of research on Vierordt’s law—Fechner’s fault? *BioRxiv*. 10.1101/450726

[CR10] Hinton SC, Rao SM (2004). “One-thousand*one* . . . one-thousand*two* . . .”: Chronometric counting violates the scalar property in interval timing. Psychonomic Bulletin & Review.

[CR11] Jazayeri M, Shadlen MN (2010). Temporal context calibrates interval timing. Nature Neuroscience.

[CR12] Jones MR, Mcauley JD (2005). Time judgments in global temporal contexts. Perception & Psychophysics.

[CR13] Lapid E, Ulrich R, Rammsayer T (2008). On estimating the difference limen in duration discrimination tasks: A comparison of the 2AFC and the reminder task. Perception & Psychophysics.

[CR14] Levitt H (1971). Transformed up-down methods in psychoacoustics. The Journal of the Acoustical Society of America.

[CR15] Moscatelli A, Mezzetti M, Lacquaniti F (2012). Modeling psychophysical data at the population-level: The generalized linear mixed model. Journal of Vision.

[CR16] Nash JC, Varadhan R (2011). Unifying optimization algorithms to aid software system users: Optimx for *R*. Journal of Statistical Software.

[CR17] Petzschner FH, Glasauer S (2011). Iterative Bayesian estimation as an explanation for range and regression effects: A study on human path integration. Journal of Neuroscience.

[CR18] Petzschner FH, Glasauer S, Stephan KE (2015). A Bayesian perspective on magnitude estimation. Trends in Cognitive Sciences.

[CR19] R Core Team. (2018). R: A language and environment for statistical computing [Computer software]. R Foundation for Statistical Computing. https://www.R-project.org/

[CR20] Raviv O, Ahissar M, Loewenstein Y (2012). How recent history affects perception: The normative approach and its heuristic approximation. PLoS Computational Biology.

[CR21] Revelle, W. (2020). psych: Procedures for psychological, psychometric, and personality research [Computer software]. https://CRAN.R-project.org/package=psych

[CR22] Roberts S, Pashler H (2000). How persuasive is a good fit? A comment on theory testing. Psychological Review.

[CR23] Shi Z, Church RM, Meck WH (2013). Bayesian optimization of time perception. Trends in Cognitive Sciences.

[CR24] Taatgen N, van Rijn H (2011). Traces of times past: Representations of temporal intervals in memory. Memory & Cognition.

[CR25] Taatgen NA, van Rijn H, Anderson J (2007). An integrated theory of prospective time interval estimation: The role of cognition, attention, and learning. Psychological Review.

[CR26] van Rijn H (2016). Accounting for memory mechanisms in interval timing: A review. Current Opinion in Behavioral Sciences.

[CR27] von Vierordt, K. (1868). *Der Zeitsinn nach Versuchen* [The experimental study of the time sense]. Laupp. https://books.google.nl/books?id=uLtbAAAAcAAJ

[CR28] Wagenmakers E-J (2007). A practical solution to the pervasive problems of p values. Psychonomic Bulletin & Review.

[CR29] Wiener M, Thompson JC, Coslett HB (2014). Continuous carryover of temporal context dissociates response bias from perceptual influence for duration. PLoS ONE.

